# Prediction of Fertilization Disorders in the *In Vitro* Fertilization/Intracytoplasmic Sperm Injection: A Retrospective Study of 106,728 Treatment Cycles

**DOI:** 10.3389/fendo.2022.870708

**Published:** 2022-04-20

**Authors:** Tian Tian, Lixue Chen, Rui Yang, Xiaoyu Long, Qin Li, Yongxiu Hao, Fei Kong, Rong Li, Yuanyuan Wang, Jie Qiao

**Affiliations:** ^1^ Center for Reproductive Medicine, Department of Obstetrics and Gynecology, Peking University Third Hospital, Beijing, China; ^2^ National Clinical Research Center for Obstetrics and Gynecology, Peking University Third Hospital, Beijing, China; ^3^ Key Laboratory of Assisted Reproduction, Peking University, Ministry of Education, Beijing, China; ^4^ Beijing Key Laboratory of Reproductive Endocrinology and Assisted Reproductive Technology, Peking University Third Hospital, Beijing, China

**Keywords:** *in vitro* fertilization, low fertilization, failed fertilization, prediction model, anti-Mullerian hormone (AMH)

## Abstract

**Purpose:**

This study aimed to develop a risk prediction of fertilization disorders during the *in vitro* fertilization/intracytoplasmic sperm injection (IVF/ICSI).

**Methods:**

A retrospective study was performed with 106,728 fresh embryo IVF/ICSI cycles from 2009 to 2019. Basic characteristics of patients, clinical treatment data, and laboratory parameters were involved. The associations between the selected variables and risks for low fertilization rate (LFR) and total fertilization failure (TFF) were investigated. Ordinal logistic regression and the receiver operating characteristic curves (ROCs) were used to construct and evaluate the prediction models.

**Results:**

A total of 97,181 controls, 4,343 LFR and 5,204 TFF cases were involved in this study. The model based on clinical characteristics (the ages of the couples, women’s BMI, types of infertility, ART failure history, the diminished ovarian reserve, sperm quality, insemination method, and the number of oocytes retrieved) had an AUC of 0.743 for TFF. The laboratory model showed that primary infertility, ART failure history, minimal-stimulation cycle/natural cycle, numbers of oocyte retrieved < 5, IVF, and Anti-Mullerian hormone (AMH) level < 1.1ng/ml are predictors of TFF, with an AUC of 0.742.

**Conclusion:**

We established a clinical and a laboratory prediction model for LFR/TFF. Both of the models showed relatively high AUCs.

## Background

During *in vitro* fertilization (IVF) and intracytoplasmic sperm injection (ICSI), fertilization disorders are important challenges in clinical practice. Fertilization disorders include a low fertilization rate (LFR, the fertilization rate < 25%) and total fertilization failure (TFF, the fertilization rate = 0%) (1). Fertilization disorders occur from 10% to 20% in the IVF cycle, while from 3% to 5% in the ICSI cycles (2, 3). The fertilization failure events could result in no or a decreased number of embryos. They could be an extremely stressful experience and entail a heavy economic burden to couples undergoing treatment and clinical physicians. Therefore, identifying and screening potential risk factors of fertilization failure during the IVF/ISCI process could help to improve the success of the treatment.

Fertilization results from sperm-egg fusion, allowing the two gametes to fuse and create the zygote (4). The fertilization process includes a series of complex processes of strictly orderly, including the oocyte and sperm growth and maturation, zona pellucida binding, sperm capacitation, gamete fusion, oocyte activation, and so on (5). Therefore, several factors may contribute to the occurrence of fertilization failure in the IVF/ICSI treatment. Previous studies reported that disrupted genetic and epigenetic patterns (6, 7), clinical characteristics (e.g., women’s age and duration of infertility, etc.) (8), laboratory bio-markers such as follicle-stimulating hormone (FSH), Anti-Mullerian hormone (AMH) (9, 10), number and quality of oocytes, as well as the semen parameters (11, 12) could affect the fertilization rate during IVF/ICSI. Up to date, there have been a few studies reporting the prediction model for fertilization disorders such as LFR and TFF in the European population and Turkey population (13–15). The sample sizes of these studies were relatively small. And the previous studies only focused on a few factors such as female smoking, male age, number of available oocytes, and sperm parameters. Besides, all the reported prediction models for fertilization disorders in IVF/ICSI had limited predictive accuracy (16). Therefore, no prediction model has been widely used in routine clinical practice yet.

In this study, we performed a retrospective study based on an extensive clinical database that involved 106,728 IVF/ICSI cycles from 2009 to 2019. We aimed to thoroughly investigate the potential risk factors of LFR and TFF, and further develop a prediction model for LFR/TFF, thus helping to provide the basis for the improvement of the success of IVF/ICSI in clinical practice.

## Methods

### Study Population

**Figure 1:**
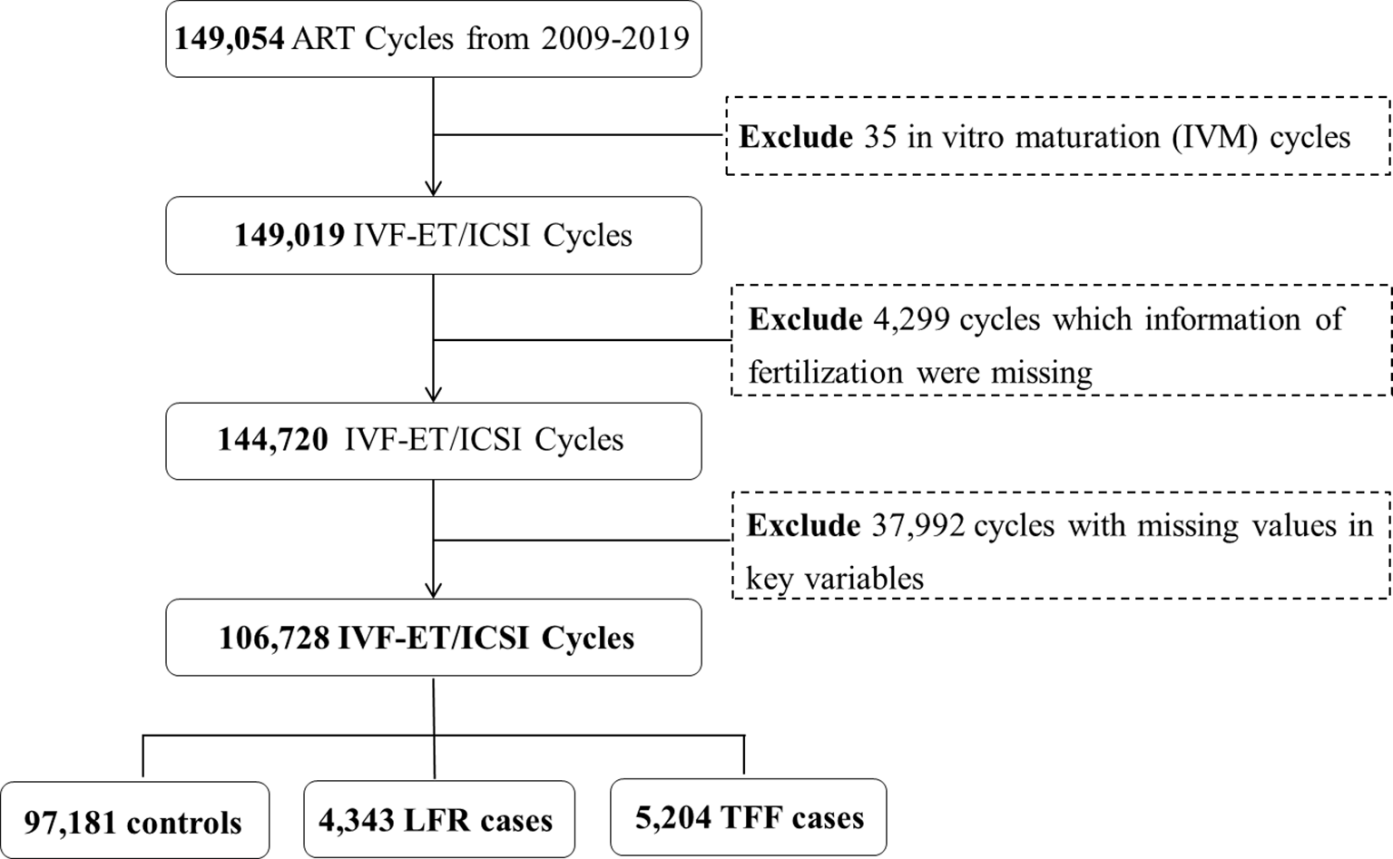
The flow chart of the participants’ selection.

The data of the participants were collected from the couples who underwent assisted reproductive technology (ART) treatment from 2009 to 2019 at the Center for Reproductive Medicine, Peking University Third Hospital, which is one of the largest reproductive health centers in China. The center has established a computer-based patient record system and recorded the patients’ information during ART cycles. We initially involved 149,054 fresh embryo ART cycles that excluded artificial insemination by donors. 4,299 cycles lacking the information of fertilization outcome and 37,992 cycles with too many missing values of the critical variables were excluded. The IVF and ICSI cycles were included and each cycle only has one insemination method (IVF or ICSI). Finally, a total of 106,728 IVF/ICSI cycles were involved in the present study (**Figure 1**).

The information of each IVF/ICSI cycle was collected, including basic characteristics of the participants [age, race, occupation, body mass index (BMI), etc.], history of diseases, the type of infertility, laboratory indicators [six test items for reproductive hormones of women including follicle-stimulating hormone (FSH), estradiol (E2), progesterone (P), luteinizing hormone (LH), testosterone (T) and prolactin (PRL), and anti-mullerian hormone (AMH)], and the variables related to the treatment process.

### Ethical Considerations

This study was approved by the Ethics Committee of Peking University Third Hospital (No. IRB00006761-M2020004). All procedures followed were in accordance with the ethical standards of the responsible committee on human experimentation and with the Helsinki Declaration of 1964 and its later amendments. Informed consent was obtained from all patients for being included in the study.

### Study Outcomes

The outcomes were defined according to the Chinese Society of Reproductive Medicine (CSRM) consensus on crucial indicators for quality control in ART clinical operation (17). IVF fertilization rate was calculated as the number of oocytes with two pronuclei/number of all collected oocytes*100. The fertilization rate of ICSI was calculated as the number of oocytes with two pronuclei/number of all collected oocytes in the MII period*100. TFF was defined as a cycle resulting in no fertilized oocytes, while LFR was defined as a cycle with a fertilization rate < 25% (18, 19).

### Prediction Models

Two prediction models were developed, including a clinical model that contains the clinical variables based on medical knowledge and availability in clinical practice, and a laboratory model that contains laboratory markers of women’s reproductive hormones.

#### Clinical Model

In this model, we involved the entire 106,728 cycles. 75% of the cycles were randomly selected as the training dataset. Predictors included the following three sections: 1) female information including age, BMI (kg/m^2^), tubal factors, uterine disorders, hyperprolactinemia, ovulatory disorders, ovarian cyst surgery, diminished ovarian reservation, and endometriosis; 2) male information including age, BMI (kg/m^2^), sperm quality [normal, oligoaasthenozoospermia (OAZ), severe OAZ, and azoospermia,which was diagnosed by WHO laboratory manual for the examination and processing of human semen[R/OL]. 5th ed (20).]. The sperm preparation and measurement methods during the past ten years were consistent. 3) the variables obtained during IVF/ICSI treatment including the type of infertility (primary or secondary), the ART failure history (including the previous failure to fertilization or failure to achieve pregnancy after ART), ovulating induction methods (natural cycle, minimal-stimulation cycle, and stimulation cycle), antral follicle count in the ovary (AFC, categorized into >12, 5-12, <5), number of retrieved oocytes (categorized into <5, 5-20, >20), insemination method (IVF and ICSI). All of the mentioned predictors were involved in the model by categorical variables. The detailed information of those variables is shown in **Table S1**. Further, 25% of the cycles were used as a validating dataset to validate the prediction model.

#### Laboratory Model

The AMH level is vital to the IVF outcomes, including fertilization rate (21), thus developing a predictive model containing AMH and other laboratory indicators is crucial to clinical practice. In this study, we used a sub-population containing 22,230 cycles with AMH information to develop a laboratory model because the test of AMH has been applied in clinical practice in recent years. 75% of the cycles were randomly selected as a training set. Besides all the mentioned variables in the clinical model, this model also included the laboratory indicators FSH (mIU/mL), E2 (pmol/L), P (nmol/L), and AMH (ng/mL). The indicators were divided into categories by reference range based on previous studies (22, 23). Detailed information is shown in **Table S1**. Similar to the analysis of the clinical model, the rest of 25% of participants were used to validate the model.

### Statistical Analyses

Considering the continuous variables were not normally distributed, the median and interquartile ranges were used to summarize the variables. Absolute frequencies and percentages were used to represent the categorical variables. Distributions of variables in control, LFR, and TFF groups were compared by Pearson’s chi-square test.

We randomly selected 75% of controls, TFF, and LFR cycles as training sets to develop the prediction models. Before establishing the model, the collinearity of the predictors was examined by the Spearman correlation test. In the training set, ordinal logistic regressions were applied to identify the predictors for LFR and TFF. The selection of the predictors used the stepwise selection method based on the Akaike information criterion (AIC). The odds ratios and their 95% confidence interval (ORs, 95%CIs) were calculated to show the associations between each predictor and the risks for LFR/TFF. We used the remaining 25% of the dataset to evaluate the discrimination of the models. The receiver operating characteristic curve (ROC) and the area under curve (AUC) were calculated. ROC shows the model sensitivity and 1-specificity, while the AUC value refers to the ability of the model to classify research objects correctly. The cut-off point of the ROC was also calculated to obtain the sensitivity and specificity of the model.

All statistical tests were two-sided, and a *P*-value < 0.05 was considered statistically significant. All analyses in this study were performed using R (version 4.1.0). The development and validation of the prediction models were implemented by using a series of R packages, including “MASS”, “caret”, “ggplot2”, “pROC”, and other R Core Teams.

## Results

### a. Characteristics of Participants

This study initially included 149,054 IVF/ICSI cycles. After excluding 35 cycles undergoing other insemination methods (e.g. *in vitro* maturation, etc.), 4,299 cycles without fertilization information and 37,992 cycles with missing values in key predictor variables, 106,728 cycles were involved in final analyses, including 97,181 controls, 4,343 LFR and 5,204 TFF cases (**Figure 1**).

The baseline characteristics of participants are shown in **Table 1**. 34.1% of the women aged ≥ 35 years. The frequencies of women with overweight or obesity, fallopian tube disorders, uterine disorders, ovulatory disorders, diminished ovarian function, and endometriosis were 29.8%, 21.4%, 7.5%, 13.1%, 9.7%, and 5.5%, respectively. 5.6% of the husbands were older than 45 years old. 63.7% of the husbands had overweight or obesity problems. 39.5% of the men had OAZ or severe OAZ, while 7.3% of them had azoospermia. Besides, the frequency of primary infertility was 54.9%, and the frequency of ART failure history was 34.3%. The frequency of 12.6% of the number of retrieved oocytes < 5. 55.8% of the cycles were IVF cycles, while the rest of 44.2% were ICSI cycles.

**Table 1:** Baseline characteristics of the study population.

	Characteristics	Levels	Overall (N=106,728)	
			N	%
**Female**				
	**Age (y)**	< 35	70307	65.9
		≥ 35	36421	34.1
	**BMI (kg/m^2^)**	18.5-24.0	66519	62.3
		< 18.5	8426	7.9
		24.0-28	23505	22
		≥ 28	8278	7.8
	**Fallopian tube disorders**		22877	21.4
	**Uterine disorders**		8048	7.5
	**Hyperprolactinemia**		424	0.4
	**Ovulatory disorder**		13929	13.1
	**Ovarian cyst surgery**		305	0.3
	**Diminished ovarian function**		10317	9.7
	**Endometriosis**		5821	5.5
**Male**				
	**Age (y)**	< 45	100746	94.4
		≥ 45	5982	5.6
	**BMI (kg/m^2^)**	< 18.5	1514	1.4
		18.5-24.0	37271	34.9
		24.0-28	45226	42.4
		≥ 28.0	22717	21.3
	**Ejaculatory dysfunction**		149	0.1
	**Teratozoospermia**		4680	4.4
	**Sperm quality**	Normal	56832	53.2
		Oaz	38709	36.3
		Severe OAZ	3402	3.2
		Azoospermia	7785	7.3
**ART**				
	**Infertility**	Primary	58604	54.9
		Secondary	48124	45.1
	**ART failure history**	No	70079	65.7
		Yes	36649	34.3
	**Ovulation induction protocol**	Stimulation cycle	101431	95
		Minimal-stimulation cycle	4645	4.4
		Natural cycle	652	0.6
	**Antral follicle count**	>12	61834	57.9
		5-12	36509	34.2
		< 5	8385	7.9
	**Number of Retrieved oocytes**	≥ 20	18487	17.3
		5-20	74756	70
		< 5	13485	12.6
	**Insemination method**	ICSI	47176	44.2
		IVF	59552	55.8

*OAZ, oligoaasthenozoospermia.

### b. The Associated Factors of Fertilization Disorders

As **Table 2** describes, the frequencies of the female with BMI ≥ 24.0 kg/m^2^, history of uterine disorders, history of endometriosis, ART failure history, and husband BMI ≥ 24.0 kg/m^2^, were significantly higher in controls than in LFR and TFF. On the contrary, in the TFF group, the frequencies of women aged ≥ 35, women with diminished ovarian, natural cycles, AFC < 5, and primary infertility, and husband age ≥ 45, were significantly higher (all *Ps* < 0.05).

**Table 2:** Comparison of distributions of the predictor in control, low fertilization rate, and total fertilization failure groups.

		Level	Control	LFR	TFF	*P*
			(N = 97,181)	(N = 4,343)	(N = 5,204)	
			n (%)	n (%)	n (%)	
**Female**						
	**Age (y)**	<35	64616 (66.5)	2921 (67.3)	2770 (53.2)	<0.001
		≥ 35	32565 (33.5)	1422 (32.7)	2434 (46.8)	
	**BMI (kg/m^2^)**	<18.5	7708 (7.9)	326 (7.5)	392 (7.5)	0.015
		18.5-24.0	60681 (62.4)	2682 (61.8)	3156 (60.6)	
		24-28	21321 (21.9)	969 (22.3)	1215 (23.3)	
		≥ 28.0	7471 (7.7)	366 (8.4)	441 (8.5)	
	**Fallopian tube disorders**	No	76302 (78.5)	3412 (78.6)	4137 (79.5)	0.244
		Yes	20879 (21.5)	931 (21.4)	1067 (20.5)	
	**Uterine disorders**	No	89967 (92.6)	3999 (92.1)	4714 (90.6)	<0.001
		Yes	7214 (7.4)	344 (7.9)	490 (9.4)	
	**Hyperprolactinemia**	No	96795 (99.6)	4327 (99.6)	5182 (99.6)	0.915
		Yes	386 (0.4)	16 (0.4)	22 (0.4)	
	**Ovulatory disorder**	No	84398 (86.8)	3764 (86.7)	4637 (89.1)	<0.001
		Yes	12783 (13.2)	579 (13.3)	567 (10.9)	
	**Ovarian cyst surgery**	No	96902 (99.7)	4330 (99.7)	5191 (99.8)	0.874
		Yes	279 (0.3)	13 (0.3)	13 (0.2)	
	**Diminished ovarian function**	No	88402 (91.0)	4031 (92.8)	3978 (76.4)	<0.001
		Yes	8779 (9.0)	312 (7.2)	1226 (23.6)	
	**Endometriosis**	No	91990 (94.7)	4083 (94.0)	4834 (92.9)	<0.001
		Yes	5191 (5.3)	260 (6.0)	370 (7.1)	
**Male**						
	**Age (y)**	< 45	91938 (94.6)	4141 (95.3)	4667 (89.7)	<0.001
		≥ 45	5243 (5.4)	202 (4.7)	537 (10.3)	
	**BMI (kg/m2)**	< 18.5	1393 (1.4)	62 (1.4)	59 (1.1)	0.084
		18.5-24.0	33992 (35.0)	1485 (34.2)	1794 (34.5)	
		24.0-28	41083 (42.3)	1848 (42.6)	2295 (44.1)	
		≥ 28.0	20713 (21.3)	948 (21.8)	1056 (20.3)	
	**Ejaculatory dysfunction**	No	97039 (99.9)	4339 (99.9)	5201 (99.9)	0.173
		Yes	142 (0.1)	4 (0.1)	3 (0.1)	
	**Teratozoospermia**	No	92942 (95.6)	4130 (95.1)	4976 (95.6)	0.232
		Yes	4239 (4.4)	213 (4.9)	228 (4.4)	
	**Sperm quality**	Normal	51519 (53.0)	2321 (53.2)	2992 (57.3)	
		OAZ	35270 (36.3)	1521 (35.0)	1918 (36.9)	<0.001
		Severe OAZ	3141 (3.2)	162 (3.7)	99 (2.2)	
		Azoospermia	7251 (7.5)	339 (8.0)	195 (3.7)	
**ART**						
	**Types of Infertility**	Primary	53107 (54.6)	2553 (58.8)	2944 (56.6)	<0.001
		Secondary	44074 (45.4)	1790 (41.2)	2260 (43.4)	
	**ART failure history**	No	64122 (66.0)	2824 (65.0)	3133 (60.2)	<0.001
		Yes	33059 (34.0)	1519 (35.0)	2071 (39.8)	
	**Ovulation induction protocol**	Stimulation cycle	92769 (95.5)	4216 (97.1)	4446 (85.4)	<0.001
		Minimal-stimulation cycle	3865 (4.0)	111 (2.6)	669 (12.9)	
		Natural cycle	547 (0.6)	16 (0.4)	89 (1.7)	
	**Antral follicle count**	< 5	7060 (7.3)	226 (5.2)	1099 (21.1)	<0.001
		5-12	33854 (34.8)	1527 (35.2)	1128 (21.7)	
		> 12	56267 (57.9)	2590 (59.6)	2977 (57.2)	
	**Number of retrieved oocytes**	≥ 20	11158 (11.5)	1 (0.0)	2326 (44.7)	<0.001
		5-20	17404 (17.9)	767 (17.7)	316 (6.1)	
		< 5	68619 (70.6)	3575 (82.3)	2562 (49.2)	
	**Insemination methods**	ICSI	44251 (45.5)	1504 (34.6)	1421 (27.3)	<0.001
		IVF	52930 (54.5)	2839 (65.4)	3783 (72.7)	

OAZ, oligoaasthenozoospermia.

### c. Prediction Model for Fertilization Disorders: Clinical Model

In the prediction model, 75% of the participants were randomly selected as a training set. The collinearity of the predictors was examined. As **Table S2** shows, the AFC and the number of retrieved oocytes had a significant correlation (*r* = 0.51, *P* < 0.001), therefore, we only included the number of retrieved oocytes in the prediction model.

The clinical model showed that women aged ≥ 35, husbands aged ≥ 45, women’s BMI higher than 28.0, primary infertility, the ART failure history, the diminished ovarian reservation, the husband with severe OAZ and azoospermia, IVF (compared to ICSI), and the number of retrieved oocytes < 5 were associated with the increased risk for LFR and TFF (all *Ps* < 0.05) (**Table 3**).

**Table 3:** The ordinal logistic regression for fertilization disorders: the clinical model.

		β	SE	t	*P*	OR (95%CI)
**Female age (y)**						
	< 35	Ref				1.00
	≥ 35	0.072	0.029	2.507	0.012	1.10 (1.04-1.17)
**Male age (y)**						
	< 45	Ref				1.00
	≥ 45	0.170	0.051	3.329	0.001	1.18 (1.07-1.31)
**Female BMI (kg/m^2^)**						
	18.5-24	Ref				1.00
	< 18.5	0.001	0.048	0.006	0.995	1.05 (0.95-1.15)
	24.0-28.0	0.067	0.031	2.181	0.029	1.06 (1.00-1.12)
	≥ 28	0.074	0.047	1.560	0.119	1.09 (1.00-1.19)
**Types of Infertility**						
	Secondary	Ref				1.00
	Primary	0.319	0.027	11.957	0.000	1.34 (1.27-1.41)
**Uterine disorders**						
	No	Ref				1.00
	Yes	0.015	0.046	0.334	0.738	1.02 (0.93-1.11)
**Ovulatory disorder**						
	No	Ref				1.00
	Yes	-0.123	0.040	-3.151	0.002	0.90 (0.83-0.98)
**Diminished ovarian function**						
	No	Ref				1.00
	Yes	0.192	0.040	4.801	0.000	1.20 (1.11-1.30)
**Sperm quality**						
	Normal	Ref				1.00
	O/A	0.053	0.027	1.945	0.052	1.02 (0.97-1.08)
	Severe O/A	0.233	0.080	2.905	0.004	1.31 (1.12-1.52)
	Azoospermia	0.126	0.060	2.094	0.036	1.20 (1.07-1.34)
**ART failure history**						
	No	Ref				1.00
	Yes	0.081	0.027	3.057	0.002	1.07 (1.01-1.13)
**Ovulation induction protoco**l						
	Stimulation	Ref				1.00
	Minimal-stimulation	0.229	0.053	4.303	0.000	1.33 (1.20-1.47)
	Natural	0.828	0.128	6.474	0.000	2.03 (1.57-2.60)
**Number of retrieved oocytes**						
	≥ 20	Ref				1.00
	5-20	0.309	0.040	7.271	0.000	1.34 (1.24-1.44)
	< 5	1.092	0.050	21.305	0.000	2.88 (2.71-3.29)
**Insemination method**						
	ICSI	Ref				1.00
	IVF	0.680	0.030	22.516	0.000	1.97 (1.85-2.09)
**Control|LFR**		3.429	0.050	68.378	0.000	
**LFR|TFF**		4.094	0.051	79.548	0.000	

**Figure 2:**
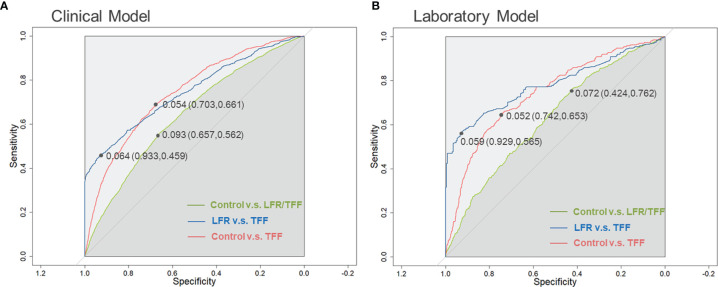
The ROC analysis of clinical and laboratory models. **(A)** ROC curves for the clinical model. This model includes women’s age, husband’s age, women’s BMI, primary infertility, the history of IVF failure, the diminished ovarian function, the husband with severe OAZ and azoospermia, IVF method, and the number of oocyte-obtained. **(B)** ROC curves for the laboratory model. This model contains predictors such as primary infertility type, ART failure history, minimal-stimulation, natural cycles, number of retrieved oocytes, IVF, and AMH level. LFR, low fertilization rate; TFF, Total fertilization failure.

The model was validated in the remaining 25% of the cycles. As a result, the prediction accuracy of the model was 91.06%. The ROCs and the AUCs are depicted in **Figure 2A**. The AUC of control versus TFF was 0.743 (95%CI: 0.729-0.757). The cut-off value of the ROC was 0.054, which had the best sensitivity of 66.1% and specificity of 70.3%. The AUC of TFF versus LFR was 0.750 (95%CI: 0.731-0.779). The cut-off value was 0.064, with the best sensitivity of 45.9% and specificity of 93.3%. We further performed the ROC analysis on the control versus the fertilization disorders (includes both TFF and LFR). The AUC was relatively low, with a value of 0.643 (95%CI: 0.632-0.655).

The distributions of characteristics between the training dataset and the testing dataset showed no significant differences (**Table S3**).

### d. Prediction Model for Fertilization Disorders: Laboratory Model

This model contained the mentioned clinical variables and the critical laboratory indicators during the treatment process, including FSH, E2, P, and AMH levels. The comparisons of the laboratory indicators’ levels are shown in **Table 4**. The median level of FSH was significantly higher in the TFF group (*P* < 0.05), while the median level of AMH was lower in the TFF group.

**Table 4:** The levels of selected biological indicators in fertilization disorder groups and controls.

		Control	LFR	TFF	*P*
		n = 20,283	n = 902	n = 1,050	
**FSH (mIU/mL)**	Median(IQR)	5.85 (3.41-7.67)	5.40 (2.64-7.38)	6.90 (4.21-9.76)	0.007
	2.8-11.3	14902 (73.5)	646 (71.6)	698 (66.5)	<0.001
	<2.8	4188 (20.6)	232 (25.7)	171 (16.3)	
	>11.3	1193 (5.9)	24 (2.7)	181 (17.2)	
**E2 (pmol/L)**	Median(IQR)	146.0 (108.0-192.0)	143.0 (106.0-188.0)	148.5 (109.0-198.0)	0.709
	73.4-1056	19307 (95.2)	863 (95.7)	1002 (95.4)	0.965
	<73.4	613 (3.0)	24 (2.7)	30 (2.9)	
	>1056	363 (1.8)	15 (1.7)	18 (1.7)	
**P (nmol/L)**	Median(IQR)	1.02 (0.72-1.41)	1.05 (0.74-1.43)	1.01 (0.74-1.43)	0.914
	< 3.6	19971 (98.5)	892 (98.9)	1034 (98.5)	0.587
	≥ 3.6	312 (1.5)	10 (1.1)	16 (1.5)	
**AMH (ng/mL)**	Median(IQR)	2.46 (1.22-4.49)	2.39 (1.36-4.36)	1.09 (0.50-2.47)	<0.001
	1.1-5	11500 (56.7)	566 (62.7)	426 (40.6)	<0.001
	<1.1	4485 (22.1)	155 (17.2)	522 (49.7)	
	>5	4298 (21.2)	181 (20.1)	102 (9.7)	

**Table 5** shows the final predictive model. In this model, primary infertility, ART failure history, minimal-stimulation cycle, and natural cycle, numbers of retrieved oocytes <5, IVF method (compared with ICSI), and AMH level < 1.1ng/ml are predictors of the risks for LFR/TFF.

**Table 5:** The ordinal logistic regression for fertilization disorders: a laboratory model.

		β	SE	t	*P*	OR (95%CI)
**Female age (y)**						
	< 35	Ref				1.00
	≥ 35	0.039	0.065	0.607	0.544	1.04 (0.92-1.18)
**Male age (y)**						
	< 45	Ref				1.00
	≥ 45	0.110	0.106	1.035	0.301	1.12 (0.90-1.37)
**Types of Infertility**						
	Secondary	Ref				1.00
	Primary	0.264	0.059	4.496	0.000	1.30 (1.16-1.46)
**Sperm quality**						
	Normal	Ref				1.00
	O/A	-0.028	0.060	-0.470	0.638	0.97 (0.86-1.09)
	Severe O/A	0.092	0.214	0.430	0.667	1.10 (0.70-1.64)
	Azoospermia	0.322	0.114	2.808	0.005	1.38 (1.10-1.72)
**Diminished ovarian function** **Diminished ovarian function**						
	No	Ref				1.00
	Yes	0.192	0.040	4.801	0.000	1.20 (1.11-1.30)
**ART failure history**						
	No	Ref				1.00
	Yes	0.111	0.058	1.921	0.050	1.12 (1.01-1.25)
**Ovulating induction protocol**						
	Stimulation cycle	Ref				1.00
	Minimal-stimulation cycle	0.390	0.102	3.807	0.000	1.48 (1.21-1.80)
	Natural cycle	0.737	0.232	3.182	0.001	2.09 (1.30-3.23)
**Number of retrieved oocytes**						
	>20	Ref				1.00
	5-20	0.434	0.105	4.152	0.000	1.54 (1.26-1.90)
	<5	1.077	0.128	8.434	0.000	2.94 (2.29-3.78)
**Insemination methods**						
	ICSI	Ref				1.00
	IVF	0.354	0.064	5.524	0.000	1.43 (1.26-1.62)
**FSH (mIU/mL)**						
	<2.8	Ref				1.00
	2.8-11.3	-0.132	0.069	-1.897	0.058	0.88 (0.77-1.01)
	>11.3	0.071	0.115	0.618	0.537	1.07 (0.86-1.34)
**AMH (ng/mL)**						
	>5	Ref				1.00
	1.1-5	0.172	0.086	2.008	0.045	1.19 (1.01-1.41)
	<1.1	0.272	0.109	2.491	0.013	1.31 (1.06-1.63)
**Control|LFR**		3.164	0.133	23.763	0.000	
**LFR|TFF**		3.841	0.136	28.342	0.000	

Similar to the clinical model, the prediction accuracy was 91.23%. The ROC analysis of control versus TFF showed an AUC of 0.742 (95%CI: 0.710-0.774), with a cut-off value of 0.052, at which point the best sensitivity was 74.2% and specificity was 65.3%. The ROC analysis of TFF versus LFR showed an AUC of 0.782 (95%CI: 0.742-0.823). The cut-off was 0.059, with the best sensitivity of 92.9% and specificity of 56.5%. The AUC of TFF/LFR versus control was 0.627 (95%CI: 0.601-0.653), with the cut-off value of 0.072 (sensitivity: 42.4%; specificity: 76.2%). (**Figure 2B**).

### e. Sensitivity Analysis

We performed several sensitivity analyses to make our results robust. First, we replaced the number of retrieved oocytes with AFC in the prediction model. As **Tables S4**, **S5** shows, the results remained consistent with the model involving the number of retrieved oocytes. But the AUCs of the models showed to be smaller than the model involved number of retrieved oocyte [AUCs and 95% CIs for clinical model: Control v.s LFR/TFF: 0.626 (0.615-0.637), LFR v.s TFF: 0.643 (0.621-0.665); Controls v.s TFF:0.684 (0.669-0.699). AUCs for laboratory model: Control v.s LFR/TFF: 0.596 (0.600-0.622), LFR v.s TFF: 0.643 (0.628-0.703); Controls v.s TFF:0.676 (0.629-0.723)]. Besides, we estimated the laboratory model involving the laboratory indicators as continuous variables. As a result, the predictors showed consistent with the model involved indicators as categories (**Table S6**) [Control v.s LFR/TFF: 0.590 (0.567-0.621), LFR v.s TFF: 0.673 (0.627-0.722); Controls v.s TFF:0.667 (0.631-0.700)].

## Discussion

Fertilization disorders, including TFF and LFR, could lead to the failure of ART. Prediction of the potential risk factors and developing useful prediction models for TFF/LFR are crucial to the success of ART treatment. Though several previous studies have investigated the risk factors for TFF/LFR, the conclusions have been uncertain; neither comprehensive predictive model with relatively high AUCs has been reported yet. In this study, we involved a large sample size with 106,728 IVF/ICSI cycles, screened and identified the critical potential predictors for TFF/LFR, and reported clinical and laboratory prediction models for TFF/LFR, thus providing new evidence and clues for the prevention of TFF/LFR.

This study identified several predictors for TFF and LFR, which could be classified into the following aspects: the female and male general characteristics, the laboratory indicators, and the ART-treatment-related variables.

Female age has been reported to be an essential factor that affects ART outcomes (24, 25). Female fertility peaks between 22 and 26 years, the age-related decrease in fertility becomes prominent at 35 years. The age of 35 is a discrete-time point after which women exhibit significantly increased risks of adverse reproductive outcomes (25), including low fertilization and low implantation rates (26). This study found that females age ≥ 35 had a higher risk for LFR/TFF, supporting the previous findings. Both high and low female BMI may increase the risks for reverse outcomes during IVF treatment (27–30). The BMI also showed interaction with female age and impacted the results of IVF (27). This study identified that the female BMI between 24.0 and 28.0 increases the risk for LFR/TFF.

In the laboratory prediction model, which involved the variables of FSH and AMH levels, female age showed no association with LFR/TFF. Given the fact that female age is associated with the level of AMH, thus the result indicates that female age may be an essential predictor of LFR/TFF when AMH is unavailable.

We identified male age and sperm quality as risk predictors of LFR/TFF in the clinical model. The influence of male age on the IVF outcomes has been controversial. One study reported that after controlling for female age using the donor oocyte model, higher male age significantly affected pregnancy outcomes and blastocyst formation rates (31). But another study reported that male age was not a factor influencing assisted reproductive techniques (32). Our data revealed that male age higher than 45 might increase LFR/TFF in the clinical model, but the effect was diminished by involving the biological indicators. Moreover, severe OAZ and azoospermia significantly increased the risk for LFR/TFF in both clinical and laboratory models. Our result suggested that sperm quality is an essential predictor of LFR/TFF in the IVF and ICSI process, which provides strong supportive evidence to the previous study (11).

The present study also revealed that several ART treatment variables were associated with a higher risk for LFR/TFF. The couples with ARTfailure history increased the risk for LFR/TFF by 1.07- and 1.12-fold in clinical and laboratory models, respectively. Ovulating induction methods contains stimulation, minimal stimulation, and natural cycles (33). The choice of ovulating induction methods has been still a controversial issue (34). This study found that compared with stimulation therapy, minimal stimulation and natural cycle are associated with the occurrence of LFR/TFF. Previous studies reported some discrepancies in the fertilization rate after ICSI and conventional IVF between studies (35, 36). Some studies showed a lower fertilization rate after ICSI (37, 38), whereas others reported no difference or a significantly higher fertilization rate after ICSI (39–42). In the present study, we found that compared with ICSI, conventional IVF had higher risk for TFF/LFR. The inconsistent of our result and previous findings may be caused by different medical centers and population, thus some systematic analysis with high quality are required to address this question. Moreover, our model found the number of retrieved oocytes was strongly associated with the fertilization rate, indicating that in clinical practice, the number of retrieved oocytes should be taken into consideration when estimate the results of ART.

Laboratory parameters including FSH, E2, P, and AMH are essential predictors of ART outcomes. Among those parameters, AMH level is one of the most important predictors of several outcomes, including ovarian response to hyperstimulation, and success of IVF therapy (21, 43). AMH inhibits the selection and maturation of follicle-stimulating hormone-dependent follicles with resultant prevention of rapid depletion of primordial follicle pool (44). Though some studies report a positive correlation between serum AMH level and fertilization rate (45), other studies suggested such a correlation could not be demonstrated (46). Therefore, the AMH level on fertilization rate has not been completely clarified yet. The present study found that the AMH level was significantly lower in TFF/LFR groups. With the decreasing of AMH level, the risk for LFR/TFF showed a significantly increased tendency. Our result illustrated the importance of AMH to the fertilization rate during IVF/ICSI treatment.

Though several prediction models of IVF/ICSI outcomes are reported, none of them has been widely used in routine clinical practice (16). One study involved 304 European TFF couples and 304 controls and evaluated the predictors of TFF. They reported that the number of available oocytes, female smoking, non-tubal factor infertility were predictors of TFF (13). Another study involved 892 couples and reported a model to predict TFF based on selected baseline characteristics (male age, number of IVF cycles, indication for IVF, and prewash total motile sperm count during fertility workup) with an AUC of 0.75 (14). The sample sizes of these previous studies were limited (sample sizes range from 304 to 892), and few of those studies validated their models or reported AUCs. Moreover, previous studies only focused on a few predictors. A prediction model that involved thorough variables during IVF/ICSI was required. This study developed clinical and laboratory models based on different types of variables using a large sample with 106,728 IVF/ICSI cycles. The clinical model indicated that female and male age, infertility type, sperm quality, ART failure history, number of retrieved oocytes, insemination method, and ovulating induction protocol are predictors of TFF, with an AUC of 0.743. The laboratory model, including AMH, showed a higher AUC (0.742 to predict TFF) than reported previous prediction models for fertilization disorders in ART.

Our study had several strengths. First, to the best of our knowledge, this is the largest sample size used in predicting the fertilization rate of ART, which could provide strong evidence and supplements to the previous findings. Second, in this study, we involved several variables, including demographic characteristics of the couple, variables in the treatment process, and laboratory indicators, which made us could thoroughly investigate potential predictors of LFR/TFF. Third, our data were collected from electronic health records, allowing us to access detailed longitudinal clinical data for large populations. And the laboratory indicators were extracted from test machines directly, thus making the data reliable and accurate.

Some limitations need to be addressed as well. First, our study is based on a single-center analysis. However, the Center for Reproductive Medicine, Peking University Third Hospital, is China’s largest reproductive health center. According to our previous statistics, over 60% of the patients come from different provinces of China, which makes our data represent the Chinese IVF/ICSI population to some extent. Second, because the diagnosis of obstructive vs. non-obstructive azoospermia has not been carried out until the second half of 2019 in our medical center. We did not include the specific subtype of azoospermia in our analysis due to the limited sample size. Third, our data were extracted from the computer-based patient record system, the sperm parameters for the insemination sample were not recorded in the system. Thus the impact of sperm parameters for the insemination sample on fertilization was absent in this study. Finally, this study does not focus on each couple, but only on individual cycles, so we could not specifically address cases with recurrent fertilization disorders, and there might be other predictors for these chronic forms of fertilization disorders. However, the readily available predictors in our model could be meaningful to the application in clinical practice.

In conclusion, this study shows that female age ≥ 35, poor sperm quality, primary infertility, ART failure history, minimal-stimulation and natural method, numbers of retrieved oocytes < 5, IVF (compared with ICSI), and low level of AMH, are associated with higher risks for fertilization disorders. We also developed a clinical model and a laboratory model based on different types of variables. Given the importance of fertilization to the success of the ART cycle, our study could help to facilitate the early identification of individuals with elevated risk for fertilization disorders and thus improve the success of ART.

## Data Availability Statement

The raw data supporting the conclusions of this article will be made available by the authors, without undue reservation.

## Ethics Statement

This study was approved by the Ethics Committee of Peking University Third Hospital (No. IRB00006761-M2020004). The patients/participants provided their written informed consent to participate in this study.

## Author Contributions

JQ and YW conceived and designed the study. LC collected the data. TT analyzed the data and drafted the manuscript. RL, RY, XL, QL, YH, and FK participated in the revision process and have approved this submission for publication.

## Funding

YW was supported by grants from Beijing Municipal Science & Technology Commission (Z191100006619086).

## Conflict of Interest

The authors declare that the research was conducted in the absence of any commercial or financial relationships that could be construed as a potential conflict of interest.

## Publisher’s Note

All claims expressed in this article are solely those of the authors and do not necessarily represent those of their affiliated organizations, or those of the publisher, the editors and the reviewers. Any product that may be evaluated in this article, or claim that may be made by its manufacturer, is not guaranteed or endorsed by the publisher.

## Abbreviations

LFR, Low Fertilization Rate; TFF, Total Fertilization Failure; ART, Assisted Reproductive Technology; IVF, *in vitro* fertilization; ICSI, Intracytoplasmic Sperm Injection; ROC, Receiver Operating Characteristic Curve; AUCs, Area Under Curve; OAZ, Oligoaasthenozoospermia; BMI, Body Mass Index; FSH, Follicle-Stimulating Hormone; AMH, Anti-Mullerian Hormone; E2, Estradiol; P, Progesterone, LH, Luteinizing Hormone, T, Testosterone; PRL, Prolactin; CSRM, The Chinese Society Of Reproductive Medicine; 95%CI, 95% Confidence Interval.
