# Supplementary material for: Prediction of Fertilization Disorders in the In Vitro Fertilization/Intracytoplasmic Sperm Injection: A Retrospective Study of 106,728 Treatment Cycles
Source: Front Endocrinol (Lausanne). 2022 Apr 20;13:870708. doi: 10.3389/fendo.2022.870708 (PMC9065263; doi:10.3389/fendo.2022.870708)
Supplement: Supplementary file 1 [file Table_1.docx]

**Table S1 The description of predictors involved in this study**

|  | **Predictors** | **Description** |
| --- | --- | --- |
| **Female** |  |  |
| **1** | **Female age (years)** | "< 35" = 0；"≥ 35" = 1 |
| **2** | **Female BMI (kg/m^2^)** | "18.5-24.0" = 0; "< 18.5" = 1; "24.0-28.0" = 2; "≥ 28" = 3 |
| **3** | **Fallopian tube disorders** | "No" = 0; "Yes" = 1 |
| **4** | **Uterine disorders** | "No" = 0; "Yes" = 1 |
| **5** | **Hyperprolactinemia** | "No" = 0; "Yes" = 1 |
| **6** | **Ovulatory disorder** | "No" = 0; "Yes" = 1 |
| **7** | **Ovarian cyst operation** | "No" = 0; "Yes" = 1 |
| **8** | **Diminished ovarian function** | "No" = 0; "Yes" = 1 |
| **9** | **Endometriosis** | "No" = 0; "Yes" = 1 |
| **Male** |  |  |
| **10** | **Male age** | "< 45" = 0；"≥ 45" = 1 |
| **11** | **Male BMI (kg/m^2^)** | "18.5-24.0" = 0; "< 18.5" = 1; "24.0-28.0" = 2; "≥ 28" = 3 |
| **12** | **Dysspermia** | "No" = 0; "Yes" = 1 |
| **13** | **Teratozoospermia** | "No" = 0; "Yes" = 1 |
| **14** | **Sperm quality** | "Normal" = 0; "Oligoaasthenozoospermia" = 1; "severe oligoaasthenozoospermia" = 2; "azoospermia" = 3 |
| **ART Treatment-related** | |  |
| **15** | **Infertility type** | "Secondary" = 0; "Primary" = 1 |
| **16** | **ART failure history** | "No" = 0; "Yes" = 1 |
| **17** | **Ovulating induction protocol** | "Stimulation cycle" = 0; "Minimal-stimulation cycle" = 1; "Natural cycle" = 2 |
| **18** | **Antral follicle count** | ">12" = 0; "5-12" = 1; "<5" = 2 |
| **19** | **Number of retrieved oocyte** | ">20" = 0; "5-20" = 1; "<5" = 2 |
| **20** | **Insemination method** | "ICSI" = 0; "IVF" =1 |
| **Laboratory indicators** | |  |
| **21** | **FSH (mIU/mL)** | "<2.8" = 0; "2.8-11.3" = 1; ">11.3" =2 |
| **22** | **E2 (pmol/L)** | "<73.4" = 0; "73.4-1056.0" = 1; ">1056.0" =2 |
| **23** | **P (nmol/L)** | "< 3.6" = 0; "≥ 3.6" = 1 |
| **24** | **AMH (ng/mL)** | ">5" = 0; "1.1-5"= 1; "<1.1" = 2 |

**Table S2. The correlation between the predictors**

| **Variables** | **Female**  **age** | **Male age** | **Female**  **BMI** | **Fertili**  **-zaiton**  **method** | **Failure**  **History**  **of ART** | **Uterine**  **dis-**  **order** | **Ovula-**  **tory dis-**  **order** | **Diminished**  **ed ovarian**  **function** | **Endome**  **triosis** | **Infertility** | **Therapy** | **Sperm**  **Quality** | **Antral**  **follicle**  **count** | **Number of**  **Oocyte-**  **obtained** |
| --- | --- | --- | --- | --- | --- | --- | --- | --- | --- | --- | --- | --- | --- | --- |
| **Female age** | 1.00* | 0.82* | 0.07* | 0.08* | 0.01 * | 0.17* | -0.13* | 0.26* | 0.02 | 0.24 | 0.18* | 0.03* | -0.41* | -0.38* |
| **Male age** | 0.82* | 1.00 * | 0.04 * | 0.04 * | 0.01 * | 0.15 * | -0.12 * | 0.21 * | 0.02* | 0.21* | 0.15* | 0.00 | -0.34 * | -0.31 * |
| **Female BMI** | 0.07 * | 0.04 * | 1.00 * | 0.02 * | -0.02 * | 0.02 * | 0.16 * | 0.00 | -0.07 * | 0.04 * | 0.03* | 0.00 | 0.08 * | -0.07 * |
| **Insemination method** | 0.08 * | 0.04 * | 0.02 * | 1.00 * | 0.05 * | 0.04 * | 0.03 * | 0.08* | 0.06 * | 0.16 * | 0.01* | 0.23 * | -0.08 * | -0.10 * |
| **ART failure history** | 0.01 * | 0.01 * | -0.02 * | 0.05 * | 1.00 * | -0.01 * | 0.05 * | -0.05 * | -0.01 * | -0.09 * | -0.02* | 0.02 * | 0.04 * | 0.03 * |
| **Uterine disorders** | 0.17 * | 0.15 * | 0.02 * | 0.04 * | -0.01 * | 1.00 * | -0.05 * | 0.06 * | 0.10 * | 0.02 * | 0.04* | 0.00 * | -0.11 * | -0.09 * |
| **Ovulatory disorder** | -0.13 * | -0.12 * | 0.16 * | 0.03 * | 0.05 * | -0.05 * | 1.00 * | -0.09 * | -0.05 * | -0.09 * | 0.05* | 0.01 * | 0.32 * | 0.11 * |
| **Diminished ovarian function** | 0.26 * | 0.21 * | 0.00 * | 0.08 * | -0.05 * | 0.06 * | -0.09 * | 1.00 * | 0.04 * | 0.05 * | 0.28* | 0.01 * | -0.35 * | -0.36 * |
| **Endometriosis** | 0.02 * | 0.02 * | -0.07 * | 0.06 * | -0.01 * | 0.10 * | -0.05 * | 0.04 * | 1.00 * | -0.06 * | 0.02* | 0.02 * | -0.11 * | -0.08 * |
| **Infertility** | 0.24 * | 0.21 * | 0.04 * | 0.16 * | -0.09 * | 0.02 * | -0.09 * | 0.05 * | -0.06 * | 1.00 * | 0.03* | 0.09 * | -0.12 * | -0.08 * |
| **Therapy** | 0.18 * | 0.15 * | 0.03 * | 0.01 * | -0.02 * | 0.04 * | 0.05 * | 0.28 * | 0.02 * | 0.03 * | 1.00* | 0.03 * | -0.15 * | -0.29 * |
| **Sperm Quality** | 0.03 * | 0.00 | 0.00 * | 0.23 * | 0.02 * | 0.00 | 0.01 * | 0.01 * | 0.02 * | 0.09 * | 0.03* | 1.00 * | -0.02 * | -0.03 * |
| **Antral follicle count** | -0.41 * | -0.34 * | 0.08 * | -0.08 * | 0.04 * | -0.11* | 0.32 * | -0.35 * | -0.11 * | -0.12 * | -0.15* | -0.02 * | 1.00 * | 0.51 * |
| **Number of retrieved oocytes** | -0.38 * | -0.31 * | -0.07 * | -0.10 * | 0.03 * | -0.09 * | 0.11 * | -0.36 * | -0.08 * | -0.08 * | -0.29* | -0.03 * | 0.51 * | 1.00 * |

* : *P* < 0.05

**Table S3.** Comparison of the distributions of predictors between the training set and testing set

|  |  | **Training-set** | **Testing-set** | ***P*** |
| --- | --- | --- | --- | --- |
|  |  | **n = 80,047** | **n = 26,681** |  |
| **Female** |  |  |  |  |
| **Age (y)** | <35 | 52729 (65.9) | 17578 (65.9) | 0.983 |
|  | ≥ 35 | 27318 (34.1) | 9103 (34.1) |  |
| **BMI (kg/m^2^)** | <18.5 | 6305 (7.9) | 2121 (7.9) | |
|  | 18.5-24.0 | 49866 (62.3) | 16653 (62.4) | 0.882 |
|  | 24-28 | 17641 (22.0) | 5864 (22.0) | |
|  | ≥ 28.0 | 6235 (7.8) | 2043 (7.7) | |
| **Fallopian tube disorders** | No | 63000 (78.7) | 20851 (78.1) | 0.057 |
|  | Yes | 17047 (21.3) | 5830 (21.9) |  |
| **Uterine disorders** | No | 74046 (92.5) | 24634 (92.3) | 0.355 |
|  | Yes | 6001 (7.5) | 2047 (7.7) |  |
| **Hyperprolactinemia** | No | 79738 (99.6) | 26566 (99.6) | 0.339 |
|  | Yes | 309 (0.4) | 115 (0.4) |  |
| **Ovulatory disorder** | No | 69584 (86.9) | 23215 (87.0) | 0.743 |
|  | Yes | 10463 (13.1) | 3466 (13.0) |  |
| **Ovarian cyst operation** | No | 79824 (99.7) | 26599 (99.7) | 0.487 |
|  | Yes | 223 (0.3) | 82 (0.3) |  |
| **Diminished ovarian function** | No | 72275 (90.3) | 24136 (90.5) | 0.421 |
|  | Yes | 7772 (9.7) | 2545 (9.5) |  |
| **Endometriosis** | No | 75730 (94.6) | 25177 (94.4) | 0.133 |
|  | Yes | 4317 (5.4) | 1504 (5.6) |  |
| **Male** | |  |  |  |
| **Age (y)** | < 45 | 75554 (94.4) | 25192 (94.4) | 0.855 |
|  | ≥ 45 | 4493 (5.6) | 1489 (5.6) |  |
| **BMI (kg/m2)** | < 18.5 | 1144 (1.4) | 370 (1.4) | 0.122 |
|  | 18.5-24.0 | 33941 (42.4) | 11285 (42.3) | |
|  | 24.0-28 | 28055 (35.0) | 9216 (34.5) |  |
|  | ≥ 28.0 | 16907 (21.1) | 5810 (21.8) |  |
| **Ejaculatory dysfunction** | No | 79938 (99.9) | 26641 (99.9) | 0.670 |
|  | Yes | 109 (0.1) | 40 (0.1) |  |
| **Teratozoospermia** | No | 76534 (95.6) | 25514 (95.6) | 0.932 |
|  | Yes | 3513 (4.4) | 1167 (4.4) |  |
| **Sperm quality** | Normal | 42591 (53.2) | 14241 (53.4) | |
|  | O/A | 28993 (36.2) | 9716 (36.4) | 0.397 |
|  | Severe O/A | 5897 (7.4) | 1888 (7.1) |  |
|  | Azoospermia | 2566 (3.2) | 836 (3.1) |  |
| **ART** | |  |  |  |
| **Types of infertility** | Primary | 44071 (55.1) | 14533 (54.5) | 0.097 |
|  | Secondary | 35976 (44.9) | 12148 (45.5) |  |
| **ART failure history** | No | 52648 (65.8) | 17431 (65.3) | 0.192 |
|  | Yes | 27399 (34.2) | 9250 (34.7) |  |
| **Ovulating induction protocol** | Stimulation cycle | 76052 (95.0) | 25379 (95.1) | 0.260 |
|  | Minimal-stimulation cycle | 3488 (4.4) | 1157 (4.3) |  |
|  | Natural cycle | 507 (0.6) | 145 (0.5) |  |
| **Antral follicle count** | < 5 | 27446 (34.3) | 9063 (34.0) | 0.567 |
|  | 5-12 | 46302 (57.8) | 15532 (58.2) |  |
|  | > 12 | 6299 (7.9) | 2086 (7.8) |  |
| **Number of retrived oocyte** | ≥ 20 | 13961 (17.4) | 4526 (17.0) | 0.139 |
|  | 5-20 | 56027 (70.0) | 18729 (70.2) |  |
|  | < 5 | 10059 (12.6) | 3426 (12.8) |  |
| **Insemination methods** | ICSI | 35366 (44.2) | 11810 (44.3) | 0.820 |
|  | IVF | 44681 (55.8) | 14871 (55.7) |  |

**Table S4.** The ordinal logistic regression for fertilization disorders: Clinical model involved in antral follicle count

|  |  | **β** | | **SE** | **t** | ***P*** | **OR (95%CI)** | |
| --- | --- | --- | --- | --- | --- | --- | --- | --- |
| **Female age (y)** | |  | |  |  |  |  |  |
|  | < 35 | Ref | |  |  |  | 1.00 | |
|  | ≥ 35 | 0.124 | | 0.029 | 4.278 | 0.000 | 1.13 (1.07-1.20) | |
| **Male age (y)** | |  | |  |  |  |  |  |
|  | < 45 | Ref | |  |  |  | 1.00 | |
|  | ≥ 45 | 0.205 | | 0.050 | 4.060 | 0.000 | 1.23 (1.11-1.35) | |
| **Female BMI** | |  | |  |  |  |  |  |
|  | 18.5-24 | Ref | |  |  |  | 1.00 | |
|  | < 18.5 | 0.048 | | 0.048 | 1.013 | 0.311 | 1.05 (0.96-1.15) | |
|  | 24.0-28.0 | 0.107 | | 0.031 | 3.469 | 0.001 | 1.11 (1.05-1.18) | |
|  | ≥ 28 | 0.166 | | 0.047 | 3.550 | 0.000 | 1.18 (1.08-1.29) | |
| **Infertility** | |  | |  |  |  |  |  |
|  | Secondary Ref | | |  |  |  | 1.00 | |
|  | Primary | 0.286 | | 0.027 | 10.733 | 0.000 | 1.33 (1.26-1.40) | |
| **Ovulatory disorder** | |  | |  |  |  |  |  |
|  | No | Ref | |  |  |  | 1.00 | |
|  | Yes | -0.076 | | 0.041 | -1.833 | 0.067 | 0.93 (0.85-1.00) | |
| **Diminished ovarian function** | |  | |  |  |  |  |  |
|  | No | Ref | |  |  |  | 1.00 | |
|  | Yes | 0.288 | | 0.040 | 7.208 | 0.000 | 1.33 (1.23-1.44) | |
| **Sperm quality** | |  | |  |  |  |  |  |
|  | Normal | Ref | |  |  |  | 1.00 | |
|  | O/A | 0.060 | | 0.027 | 2.187 | 0.029 | 1.06 (1.01-1.12) | |
|  | Severe O/A | 0.214 | | 0.081 | 2.657 | 0.008 | 1.24 (1.05-1.45) | |
|  | Azoospermia | 0.172 | | 0.059 | 2.909 | 0.004 | 1.19 (1.06-1.33) | |
| **Failure history of ART** | |  | |  |  |  |  |  |
|  | No | Ref | |  |  |  | 1.00 | |
|  | Yes | 0.074 | | 0.027 | 2.782 | 0.005 | 1.08 (1.02-1.13) | |
| **Ovulating induction protocol** | |  | |  |  |  |  |  |
|  | Stimulation | | Ref |  |  |  | 1.00 | |
|  | Minimal-stimuation | 0.456 | | 0.052 | 8.759 | 0.000 | 1.58 (1.42-1.75) | |
|  | Natural | 0.861 | | 0.131 | 6.589 | 0.000 | 2.37 (1.82-3.04) | |
| **Antral follicle count** | |  | |  |  |  |  |  |
|  | >12 | Ref | |  |  |  | 1.00 | |
|  | 5-12 | 0.174 | | 0.049 | 5.698 | 0.000 | 1.19(1.12-1.27) | |
|  | <5 | 0.586 | | 0.043 | 11.887 | 0.000 | 1.80(1.63-1.98) | |
| **Insemination methods** | |  | |  |  |  |  |  |
|  | ICSI | Ref | |  |  |  | 1.00 | |
|  | IVF | 0.693 | | 0.030 | 23.180 | 0.000 | 2.00 (1.89-2.12) | |
| **control\|LFR** |  | 2.707 | | 0.056 | 48.057 | 0.000 |  |  |
| **LFR\|TFF** |  | 3.368 | | 0.057 | 58.684 | 0.000 |  |  |

**Table S5.** The ordinal logistic regression for fertilization disorders: laboratory model involved in antral follicle count

|  |  | **β** | **SE** | **t** | ***P*** | **OR (95%CI)** |
| --- | --- | --- | --- | --- | --- | --- |
| **Female age (y)** | |  |  |  |  |  |
|  | < 35 | Ref |  |  |  | 1.00 |
|  | ≥ 35 | 0.119 | 0.065 | 1.827 | 0.068 | 1.13 (0.99-1.28) |
| **Male age (y)** | |  |  |  |  |  |
|  | < 45 | Ref |  |  |  | 1.00 |
|  | ≥ 45 | 0.178 | 0.105 | 1.705 | 0.088 | 1.20 (0.97-1.46) |
| **Infertility** | |  |  |  |  |  |
|  | Secondary | Ref |  |  |  | 1.00 |
|  | Primary | 0.287 | 0.027 | 9.430 | 0.000 | 1.34 (1.23-1.41) |
| **Sperm quality** | |  |  |  |  |  |
|  | Normal | Ref |  |  |  | 1.00 |
|  | O/A | -0.029 | 0.060 | -0.478 | 0.632 | 0.97 (0.86-1.09) |
|  | Severe O/A | 0.343 | 0.199 | 1.722 | 0.085 | 1.41 (0.94-2.05) |
|  | Azoospermia | 0.330 | 0.115 | 2.874 | 0.004 | 1.39 (1.11-1.74) |
| **Failure history of ART** | |  |  |  |  |  |
|  | No | Ref |  |  |  | 1.00 |
|  | Yes | 0.088 | 0.058 | 1.530 | 0.126 | 1.09 (0.98-1.22) |
| **Diminished ovarian function** | | |  |  |  |  |
|  | No | Ref |  |  |  | 1.00 |
|  | Yes | 0.319 | 0.081 | 3.913 | 0.000 | 1.38 (1.17-1.61) |
| **Ovulating induction protocol** | |  |  |  |  |  |
|  | Stimulation cycle | Ref |  |  |  | 1.00 |
|  | Minimal-stimulation cycle | 0.432 | 0.065 | 6.683 | 0.000 | 1.54 (1.36-1.75) |
|  | Natural cycle | 0.324 | 0.103 | 3.134 | 0.002 | 1.38 (1.13-1.69) |
| **Antral follicle count** | |  |  |  |  |  |
|  | > 12 | Ref |  |  |  | 1.00 |
|  | 5-12 | 0.074 | 0.078 | 0.954 | 0.340 | 1.08 (0.93-1.26) |
|  | <5 | 0.377 | 0.109 | 3.440 | 0.001 | 1.46 (1.18-1.80) |
| **Insemination** |  |  |  |  |  |  |
|  | ICSI | Ref |  |  |  | 1.00 |
|  | IVF | 0.432 | 0.065 | 6.683 | 0.000 | 1.54 (1.36-1.75) |
| **FSH** |  |  |  |  |  |  |
|  | <2.8 | Ref |  |  |  | 1.00 |
|  | 2.8-11.3 | -0.115 | 0.070 | -1.643 | 0.100 | 0.89 (0.78-1.02) |
|  | >11.3 | 0.154 | 0.114 | 1.349 | 0.177 | 1.17 (0.93-1.46) |
| **AMH** |  |  |  |  |  |  |
|  | >5 | Ref |  |  |  | 1.00 |
|  | 1.1-5 | 0.242 | 0.090 | 2.703 | 0.007 | 1.27 (1.07-1.52) |
|  | <1.1 | 0.420 | 0.112 | 3.733 | 0.000 | 1.52 (1.22-1.90) |
| **Control\|LFR** |  | 2.971 | 0.152 | 19.584 | 0.000 |  |
| **LFR\|TFF** |  | 3.647 | 0.154 | 23.719 | 0.000 |  |

**Table S6.** The ordinal logistic regression for fertilization disorders: laboratory model: AMH was involved as a continuous variable

|  |  | **β** | **SE** | **t** | ***P*** | **OR (95%CI)** |
| --- | --- | --- | --- | --- | --- | --- |
| **Female age (y)** | |  |  |  |  |  |
|  | < 35 | Ref |  |  |  | 1.00 |
|  | ≥ 35 | 0.120 | 0.065 | 1.851 | 0.064 | 1.13 (0.99-1.28) |
| **Male age (y)** | |  |  |  |  |  |
|  | < 45 | Ref |  |  |  | 1.00 |
|  | ≥ 45 | 0.179 | 0.104 | 1.716 | 0.086 | 1.20 (0.97-1.46) |
| **Infertility** | |  |  |  |  |  |
|  | Secondary | Ref |  |  |  | 1.00 |
|  | Primary | 0.618 | 0.059 | 6.594 | 0.000 | 1.18 (1.21-1.31) |
| **Sperm quality** | |  |  |  |  |  |
|  | Normal | Ref |  |  |  |  |
|  | O/A | -0.032 | 0.060 | -0.538 | 0.591 | 0.97 (0.86-1.09) |
|  | Severe O/A | 0.324 | 0.199 | 1.627 | 0.104 | 1.38 (0.92-2.01) |
|  | Azoospermia | 0.331 | 0.115 | 2.892 | 0.004 | 1.39 (1.11-1.74) |
| **Ovulating induction protocol** | |  |  |  |  |  |
|  | Stimulation cycle | Ref |  |  |  |  |
|  | Minimal-stimulation cycle | 0.389 | 0.101 | 3.857 | 0.000 | 1.47 (1.21-1.79) |
|  | Natural cycle | 1.145 | 0.223 | 5.142 | 0.000 | 3.14 (2.00-4.80) |
| **Diminished ovarian function** | |  |  |  |  |  |
|  | No  y | Ref |  |  |  |  |
|  | Yes | 0.356 | 0.075 | 4.748 | 0.000 | 1.43 (1.23-1.65) |
| **Number of oocyte-obtained** | |  |  |  |  |  |
|  | >20 | Ref |  |  |  |  |
|  | 5-20 | 0.037 | 0.078 | 0.466 | 0.641 | 1.04 (0.89-1.21) |
|  | <5 | 0.362 | 0.109 | 3.318 | 0.001 | 1.44 (1.16-1.78) |
| **Fertility method** | |  |  |  |  |  |
|  | ICSI | Ref |  |  |  |  |
|  | IVF | 0.430 | 0.065 | 6.658 | 0.000 | 1.54 (1.36-1.75) |
| **AMH** | | -0.061 | 0.013 | -4.726 | 0.000 | 0.94 (0.92-0.96) |
| **Control\|LFR** | | 2.553 | 0.148 | 17.254 | 0.000 |  |
| **LFR\|TFF** | | 3.229 | 0.150 | 21.530 | 0.000 |  |
